# Wait time management strategies for total joint replacement surgery: sustainability and unintended consequences

**DOI:** 10.1186/s12913-017-2568-6

**Published:** 2017-09-07

**Authors:** Marie-Pascale Pomey, Nathalie Clavel, Claudia Amar, Juan Carlos Sabogale-Olarte, Claudia Sanmartin, Carolyn De Coster, Tom Noseworthy

**Affiliations:** 1 Departement of Health Policy, Management and Evaluation, School of Public Health, University of Montreal, Montreal, Canada; 2 Health Analysis Division, Statistics Canada, Ottawa, Canada; 3 Community Health Science, University of Calgary, Calgary, Canada; 4 Community Health Services, University of Calgary, Calgary, Canada

## Abstract

**Background:**

In Canada, long waiting times for core specialized services have consistently been identified as a key barrier to access. Governments and organizations have responded with strategies for better access management, notably for total joint replacement (TJR) of the hip and knee. While wait time management strategies (WTMS) are promising, the factors which influence their sustainable implementation at the organizational level are understudied. Consequently, this study examined organizational and systemic factors that made it possible to sustain waiting times for TJR within federally established limits and for at least 18 months or more.

**Methods:**

The research design is a multiple case study of WTMS implementation. Five cases were selected across five Canadian provinces. Three success levels were pre-defined: 1) the WTMS maintained compliance with requirements for more than 18 months; 2) the WTMS met requirements for 18 months but could not sustain the level thereafter; 3) the WTMS never met requirements. For each case, we collected documents and interviewed key informants. We analyzed systemic and organizational factors, with particular attention to governance and leadership, culture, resources, methods, and tools.

**Results:**

We found that successful organizations had specific characteristics: 1) management of the whole care continuum, 2) strong clinical leadership; 3) dedicated committees to coordinate and sustain strategy; 4) a culture based on trust and innovation. All strategies led to relatively similar unintended consequences. The main negative consequence was an initial increase in waiting times for TJR and the main positive consequence was operational enhancement of other areas of specialization based on the TJR model.

**Conclusion:**

This study highlights important differences in factors which help to achieve and sustain waiting times. To be sustainable, a WTMS needs to generate greater synergies between contextual-level strategy (provincial or regional) and organizational objectives and constraints. Managers at the organizational level should be vigilant with regard to unintended consequences that a WTMS in one area can have for other areas of care. A more systemic approach to sustainability can help avoid or mitigate undesirable unintended consequences.

## Background

The Canadian healthcare system adheres to five basic principles, including: 1) Universal coverage (all residents have a right to seek insured healthcare services, based on standard methods; 2) public governance (the health insurance system in one province or territory is managed in a non-profit way by a public authority); Access (no financial obstacle or other can hinder satisfactory access to services provided by a hospital or a physician) for those insured; 4) transferability (allows an ensured person who moves or travels within Canada, or travels outside of the country, to be covered); and 5) integrality (all necessary medical services offered by hospitals and doctors are covered). This means that care provided by physicians working within healthcare organizations is entirely covered despite them being independent practitioners remunerated on a fee-for-service basis. In addition to these principles, Canada’s 10 provinces are responsible for upholding them and to manage each of their healthcare systems. However, one of these principles, access, as with many countries, has been a significant issue for many years. Unacceptably long waiting times have contributed to public concern about the viability of a single-tier, publicly funded health care system in Canada [1].

In 2003, the “First Ministers’ Accord on Health Care Renewal” expressed that all Canadians should have timely access to care, more specifically to diagnostic procedures and medical treatments [2]. In 2004, Canadian First Ministers reiterated their promise and agreed to a “10-Year Plan to Strengthen Health Care” which included an agreement by the provinces to provide wait time benchmarks and measurable wait time reductions in five priority areas (cancer, heart, diagnostic imaging, joint replacement and sight restoration) [2]. To stimulate the necessary improvements required by this plan, the Federal Government allocated $41.3 billion in new funding to the provinces and territories over the plan’s ten-year period [2], including $5.5 billion for initiatives to decrease waiting times in the provinces, and $500 million as a Medical Equipment Fund. The provincial and territorial ministries of health developed a benchmark waiting time for total hip or knee joint replacements, that they be carried out within 26 weeks [3]. In Canada, Wait times refer to the length of time it takes people to access health care services such as specialist services, diagnostics and treatment services from decision to treat until intervention.

Since there was no real strategy at the national level to improve access to total joint replacements (TJR), provinces and territories have implemented a number of Wait Time Management Strategies (WTMS) at the provincial and hospital levels, including benchmarking, IT solutions, central booking systems, clinical assessment and prioritization tools, and clinical appropriateness guidelines [4]. Although some studies have looked at WTMS implementation [5], little research has been done on the sustainability of successful WTMS at the healthcare organization (HCO) level [6, 7]. Moreover, although it is important to know the effects of policies, programs, interventions, or strategies before implementing them, to prevent potentially negative consequences and to ensure effectiveness and long-term sustainability [8, 9], there is currently no information available for Canada and elsewhere on unanticipated consequences of WTMS that could negatively affect the sustainability of these strategies [10].

Therefore, the objectives of this research are to elucidate the organizational and contextual factors that enhance or inhibit the sustainability of WTMS for TJR and to examine unintended consequences linked to the introduction of these strategies, with the aim of providing guidance to decision-makers at the provincial and regional levels, as well as HCO managers, on how they can help to sustain WTMS over the long term, while avoiding or mitigating negative consequences.

The research conclusions should be useful for Canada and other countries facing wait time issues for access to care.

## Theoretical frameworks

**Fig. 1:**
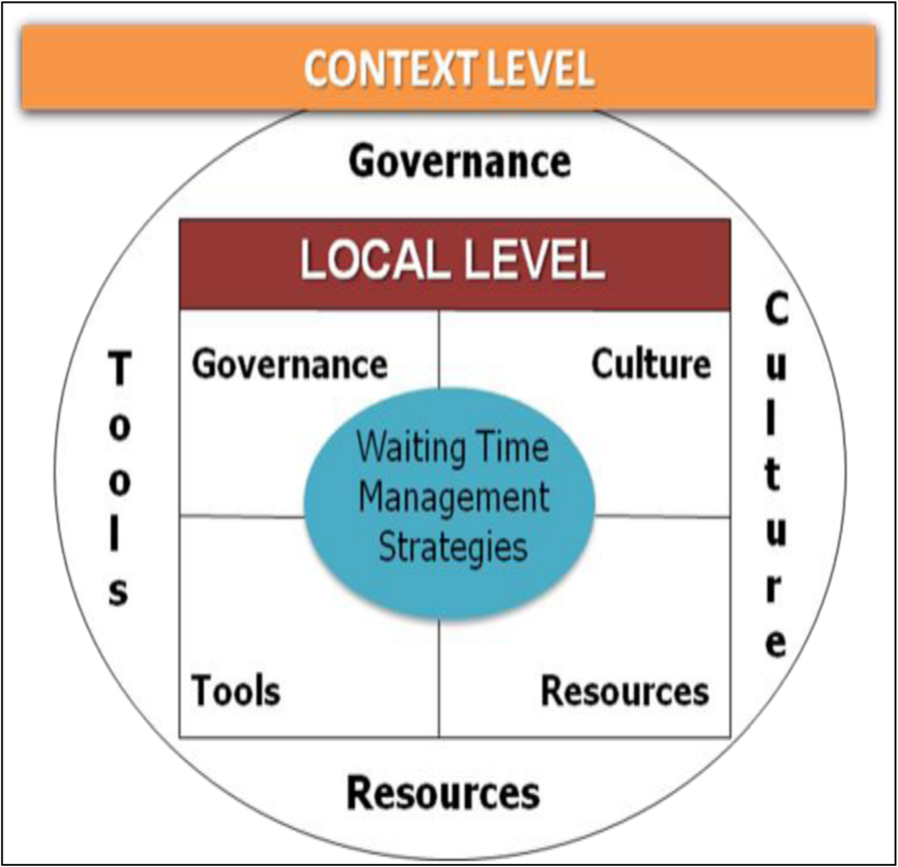
Factors that can have impact on WTMS at the local and contextual levels [36]

To analyze factors than can have an impact on WTMS sustainability at the HCO level, we used a conceptual framework developed by Pomey et al. [11], based on Parsons’ widely recognized four-quadrant model [12]. This model ensured that we captured all the dimensions of the WTMS being studied that might have contributed to sustainably achieving established wait time targets. The four dimensions we used are present at both contextual and organizational levels (Fig. 1):

1. Governance, defined as “the conduct of collective action from a position of authority” [13].
2. Cultural and leadership factors, defined as “underlying beliefs, values, norms and behaviours” including physicians’ involvement [14].
3. Methods and tools, the instruments or procedures seen as helpful in implementing a strategy.
4. Resource factors, whether human, financial, infrastructural, or informational.

In addition, to categorize unintended consequences related to implementation and sustainability of the WTMS, we developed an integrated model of consequences within HCOs, adapted from Bloomrosen et al. [15] and Rogers [16]. It presents four different types of consequences: two anticipated (goals/intended consequences and trade-offs) and two unanticipated (serendipities and negative consequences). This framework helps to distinguish between desirable and undesirable, anticipated and unanticipated, and direct and indirect consequences of WTMS. In our research, we analyzed both types of unanticipated consequences: negative consequences and serendipities (Table 1).

**Table 1:** Integrated model of unintended consequences within healthcare organizations

	ANTICIPATED (direct or indirect)	UNANTICIPATED (direct or indirect)
DESIRABLE	Goals and intended consequences	Serendipities
UNDESIRABLE	Trade-offs	Negative consequences (“Classic” unintended consequences)

Source: Bloomrosen et al. [15] and Rogers [16]

## Methods

From 2010 to 2012, we conducted an in-depth retrospective case study [17] with two embedded levels of analysis – contextual and organizational [18, 19] – of five Canadian HCOs that had implemented WTMS with various degrees of sustainability.

### Case selection

Five cases with very different WTMS sustainability results were selected using an intensity sampling approach [20, 21], defining three types of ‘intense cases’ according to a classification system inspired by Appleby et al. [22]. Based on Appleby’s proposed definitions, experts from the Western Canada Waiting List project, which include experts known in this field, have established the definitions that we ended up using to classify the cases.

- ▪ Sustainable: a WTMS that results in all patients waiting less than the 26-week benchmark for TJR, for at least 6–12 months within an 18-month period.
- ▪ Moderately sustainable: a WTMS that reduces waiting times to less than 26 weeks within an 18-month period but was not able to sustain these.
- ▪ Non-sustainable: a WTMS that fails to reduce waiting times to less than 26 weeks within an 18-month period.

We collected wait time data for the period from April 2009 to September 2010 from Canadian HCO websites and contacted HCOs to verify their status for all patients. Three organizations representing non-sustainable cases declined to participate in the study, as did one sustainable case. In the end, five organizations agreed to participate: two sustainable cases, two moderately sustainable, and one non-sustainable, whereas we had initially planned to conduct six.

### Data collection methods

In all, 37 interviews in-person were conducted for the five cases studied: eight for cases 1, 2, and 4; seven for case 3; and six for case 5. No participants declined the invitation for an interview. The interviews, which lasted up to 60 min, were conducted with a range of decision makers at the provincial/regional level, healthcare providers and managers, including quality directors, chiefs of surgery, orthopedic surgeons, front-line managers, nurses, and physiotherapists. A Semi-directed interview guide was used to do the interviews (Additional file 1). 

We consulted documentation on access and surgical volumes related to each initiative for the period from April 2009 to September 2010, as well as various sources and studies regarding the strategies. The use of multiple data sources was helpful in generating complex theories and strengthening empirical grounding [23]. The documents were used to describe the WTMS implementation processes and to verify or clarify factors cited by the interviewees. All the interviews were transcribed. In the one case where interviews were conducted in French, transcripts were translated into English. The transcripts were analyzed by two reviewers using content analysis, guided by our theoretical framework and using the software NVivo. Divergent issues were discussed until the research group reached a consensus.

### Data analysis

To compare cases, we used data reduction and presentation techniques similar to those suggested by Huberman and Miles [24]. For each case, we used all relevant documents and interview transcripts to identify the factors at the organizational and contextual levels that contributed to sustaining the WTMS. We summarized the findings for each case and sent it to the senior manager responsible for the WTMS to validate them. Their feedback and interpretation of findings was incorporated into the results.

We presented our findings at the “Taming of the Queue” conference [10, 25], attended by decision-makers responsible for WTMS in various Canadian settings. Asked to comment on our research findings, they unanimously confirmed that our findings matched their understanding as to why some organizations were able to sustain WTM while others were not. They also confirmed the positive and negative unintended consequences and agreed that they were facing the same dynamics.

## Results

This section presents the factors that influenced sustainability for each case study and examines unintended consequences at the organizational level related to WTMS. We also look at systemic/contextual factors that supported or impeded sustainability at the organizational level.

### Presentation of the five cases

A summary profile of the five cases, which represent the full range of service provision for TJR, is provided in Table 2. The factors helping/hindering WTMS and unintended consequences for each case are summarized in individual tables (Tables 3, 4, 5, 6 and 7), along with a selection of supporting verbatim quotes from interviews. Each quote is referenced using an “I” for interview, followed by the case number and the interviewee number.

**Table 2:** Case profiles

	Case 1 Atlantic Canada	Case 2 Central Canada	Case 3 Western Canada	Case 4 Central Canada	Case 5 Central Canada
Status	Non-sustainable	Moderate	Moderate	Sustainable	Sustainable
Location	Atlantic Canada	Central Canada	Western Canada	Central Canada	Central Canada
Type of HCO	Tertiary-care teaching hospital Acute medical and surgical care	Community teaching hospital Acute medical and surgical care	Secondary-care hospital Acute medical and surgical care	Tertiary-care teaching hospital Focuses on TJR	Community teaching hospital Acute medical and surgical care and long term care
Implementation date	2008	2007	2009	2007	2007
Types of WTMS	Contract with the authority and the HCO to do 500 additional orthopedic surgeries Provincial database patient access registry Central referral office (provincial Orthopedic Assessment Clinic) Pathways Healthcare Scheduling (PHS) system First available surgeon	Pathway Regional Joint Assessment Center Advanced practice physiotherapist Triage in three categories First available surgeon	Regional Central Intake (based on Patient Access Registry Tool (PART) Pre-surgical clinic and team Triage in three groups	Regional Steering Committee implementing a comprehensive hip and knee replacement program (HKRP) Patient pathway (pre-op to rehabilitation) Intake and Assessment Centre model consisting on a Central Intake Advanced practice physiotherapist	Central Intake Patient pathway (pre-op to rehabilitation) Clustering of pre-operation examinations Physical rehabilitation technicians

**Table 3:** Case 1 (Atlantic Canada) - Factors affecting WTMS sustainability and unintended consequences

Organizational factors	
Governance	- Lack of incentives to encourage staff engagement (including nurses): “*To make things work…. There have to be incentives; so the incentives could be either paid time –…scheduled into their regular time. It’s usually not above their normal time… If we can find some people that see the benefit of reducing the wait time so that the patients come in healthier, then that information will then spread. But it’s a lot of education with the nurses to get them to buy into processes. And the administrative culture is slowly changing,… it’s very difficult to get change.” (I.1.3)* - Lack of upper management involvement and of support for WTMS within the hospital
Culture	- Physicians not all engaged and cultural gap between senior and junior surgeons: “*We have some people that have been doing surgery here for a very long time – twenty-five plus years – and they like it the way it was, and it’s very difficult to change. Our new surgeons are sold on technology, transparency… using people to their maximum potential;… employing a physiotherapist to do some assessments,… using the nurses to do that kind of thing, and have the surgeon do surgery. So we have two different camps right now… it’s a culture thing, it’s a generational gap; we do meet resistance from… the old boys’ club” (I.1.2*).
Methods and tools	- Public website informing patients and families about TJR wait times (within hospitals in the region) - Computerized Pathway Healthcare Scheduling (PHS) system used for booking and registration - Discharge abstract database containing retrospective data on patients - Lack of standardization of the referral process resulting in delays in the referral process: *“We didn’t catch that it would have been necessary to have a standardized process for our referral process from the beginning”. (I.1.5)*
Resources	- Increased resources allocated for renovation of assessment clinic, booking and registration system, clinical staff, patient information website, increased OR time - Nursing shortages due to budget constraints - Physicians competing with other specialties for OR time and access
Main contextual factors	
- Insufficient funding considering large proportion of population with arthritis in the province: “*I’m always trying to meet the target... We’ve met the target, but there’s more coming in through the door, and... we have a very high arthritis population... So it’s not a surprise that as we become older there’s going to be more people waiting. So…if the expectation is to get down to that 180 days or 182 days – whatever people dispute that it is – then there’s got to be some capacity”. (I.1.3)*	
Unintended consequences	
Serendipities	- Development of new educational programs for others patients: - Model for improvement in other areas of specialization: *So this strategy has helped us evaluate other programs; because spine has a huge wait time, and 95% of people that refer for spine surgery don’t require spine surgery…. There’s been positive spin-offs from the strategy for other surgeries. (I.1.2)*
Negative consequences	- Increased wait times resulting from public awareness and patient’s preferences (times and surgeons): “*Now people are going to start reporting their wait time by province, by district, or by area –you could end up getting more referrals because your waits are lower…. if it’s out on the public website, all the family docs can look at it…. So it starts becoming referring to a hospital* versus *referring to a surgeon…. So you get a dump with a lot of referrals pretty quick, and if you get a huge amount coming in, … you’re probably not going to be meeting your benchmark anymore”. (I.1.4)*

**Table 4:** Case 2 (Central Canada) - Factors affecting WTMS sustainability and unintended consequences

Organizational factors	
Governance	- Good support from middle managers in helping physicians find solutions and implement them - Leadership by a non-clinical director of surgical services: “*I’m not a nurse or a surgeon, I came in and said, ‘Well, this doesn’t really make sense, we need to work together,’ so...over the years we’ve really improved that*” (I.2.1). - Shared leadership between APPs and surgeons
Culture	- Tension between different types of physicians and between physicians and OR nurses/anesthesiologists - Lack of culture of collaboration among nurses and surgeons *Physicians are independent practitioners, they’re not employees of the hospital, so unless you’re partners with them and doing some of these creative collaborative things and getting them on your side, you can maybe get some traction but...otherwise...there’s no incentive. (I.2.1)*
Methods and tools	- Province-wide wait time system software (iPort) that provides self-service reporting of wait times, as previously existing system was inadequate: “*They’re not really big fans of the wait times system or the requirements under them, so the quality of the data reporting was poor, and there were some accuracy issues”* (I.2.9). - Provincial metric: *The provincial metric will remain, as far as I know, the 90th percentile. That’s the direction they’re going on all of the indicators, because the political reason for it is that the longest-waiting person is waiting for service and that’s what really needs to be measured. (I.2.3)*
Resources	- Inefficient utilization of OR rooms, - Insufficient staffing (nurses and anesthesiologists) - Inefficient bed management and utilization
Main contextual factors	
- Health region support for professionals to improve data collection and interpretation. - Latitude in HCO activities to meet health region target: *“We don’t micromanage the providers and tell them specifically what activities to undertake. They’re basically required to meet the accountabilities in their agreements so... there is an accountability agreement there that specifies that …182 days is the number for wait times, and basically whatever reasonable activities they need to undertake to do, that is up to them.” (I.2.2)* - Negotiation between HCO and health region authority on indicators used to justify funding: *“In an ideal steady-state environment, if there was an increase in volumes, you would see wait times drop, but in many cases there’s actually an inverse relationship because… the wait time isn’t measured until the procedure is done. So I often say on a lot of these indicators that it’s going to get worse before it gets better.” (I.2.6)*	
Unintended consequences	
Serendipities	- Model for other programs to improve referral processes and dialogue with GPs: “*This is a good model, because it helps to ensure that there is a good distribution of who is receiving the referrals… Now the lessons learned out of ortho are certainly being applied across the board to many areas.” (I.2.4)*
Negative consequences	- Increases in wait time due to double referrals - Increase in wait list due to patients preferences: “*Dr. Y… is an excellent surgeon, but Dr. X has a better reputation, for whatever reason, in the community. So his [Dr. Y’s] wait time is usually around 160–170 days, whereas Dr. X’s real wait time is usually…closer to 230 days. So they say, ‘Well, for another two months,… I’ll just wait for Dr. X, it’s not a big deal.’ But to the Ministry… it’s hard for us to capture that.” (I.2.3).* - Increase in nosocomial infections

**Table 5:** Case 3 (Western Canada) - Factors affecting WTMS sustainability and unintended consequences

Organizational factors	
Governance	- High support and governance from RHA level - Hospital mainly dedicated to elective orthopedic surgery and implementation of a two-room model - Development of a centralized intake process and adjustment of referrals distribution: “*We’ve been going through call distribution strategy because [Hospital X] has gotten to a point where they’re actually refusing referrals because there’s no way they can see those patients in a timely manner…So we spend a lot of time focused on redistribution and centralized intake…We were calling patients and literally offering patients another surgeon or another date and another time.” (I.3.2)*
Culture	- Physician engagement, leadership and innovative culture within the Hip and Knee Institute - Lack of common goals and values related to WTM among surgeons across all sites: “*There always seems to be a dollar for any kind of improvement and sustaining that; … making the money the reason you make change,… to influence people’s participation, but it shouldn’t be the reason why we’re making that change, and getting people aligned on the why we need to make a change has been very challenging. I don’t think people have embodied the values, and to me that’s one of the challenges on why we can’t sustain this, because we don’t have people aligned on the goal.” (I.3.1)*
Methods and tools	- RHA developed Patient Access Registry Tool (PART) to reconcile and monitor patient wait lists - Standardized common referral form for surgeons: “*So this is the flow chart for the common intake process. So it starts with the standardized referral form –either paper… There’s actually an online version called bridging generalist to specialist care: BGSC”* (*I.3.4*)
Resources	- Increased human resource staffing: clinical assistants for OR, staff for pre-habilitation clinic - Resources not allocated specifically to HR: *“There were not clearly specific resources allocated to staffing, but a global envelope dedicated to increase case volumes, so those have not been sidelined directed resources. For instance, they’ve used the pre-hab clinics, which were established, and the resources associated with them, to support some of the activity as well. The people doing the work have been the same people who existed prior and post, they’re not tied to the funding.” (I.3.1)*
Unintended consequences	
Main contextual factors	
- RHA funding to increase volumes of TJR - Strong leadership at both provincial and RHA level	
Serendipities	- Model for other programs and specialties
Negative consequences	- Increases in wait time due to patient and GP misunderstanding of the referral process and patient’s preferences (times and surgeons): “*Basically, given the validation reference we’ve made, when repeated in many other areas, we see a range of between 20% up to 50 and 60% of inappropriate referrals being made and inappropriate patients sitting on wait lists.” (I.3.4)* - Increases in wait time due to the high reputation of the Institute: “*Then once you start decreasing that wait, you find all these undiscovered bottlenecks. … it seemed like the whole city thought that the only place you could have your hip or knee done was here at [Hospital X], so… we got backlogged again…, started actively redirecting uncomplicated consults to surgeons with the shortest waitlist in town.” (I.4.2)* - Changes to strategy impacted patient satisfaction (positively and negatively, depending on timing) and led to staff exhaustion

**Table 6:** Case 4 (Central Canada) - Factors affecting WTMS sustainability and unintended consequences

Organizational factors	
Governance	- Strong leadership from hospital CEO and upper management - Surgeons, staff, and middle and top managers aligned toward the same goal: to treat people in as excellent a way as possible - Progressive implementation of an APP (advanced practice physiotherapist) model: “*It’s like you’ve done your homework up front, you have a plan, you [do] exactly what you say, small steps, and ... we definitely used plenty of study methodology. So we would do small cycles of change, and what did work, we built on bigger, but if it didn’t, you hadn’t invested so much that you couldn’t change and do something different and tweak it differently.” (I.4.6)* - Committees to coordinate strategy and to sustain ongoing work
Culture	- Hospital mainly dedicated to elective orthopedic surgeries - Culture of innovation shared by all the professionals, staff and managers (middle and top) - Culture of quality improvement and interdisciplinary teams, stability, cohesiveness and unified culture: “*There are a lot of old-timers, as we call each other…. We have a great team here and that, I really believe, has been another… critical success factor, because it’s a very cohesive team. There’s input from everybody, everyone takes part in [the] initiative…. So I think that the team works very well. [When] we have a solid team like that, you’re at an excellent starting point, right?” (I.2.8)*
Methods and tools	- Central intake clinic and referral tracking system (provincial wait time information system) - Development of a standardized request form for consultation - Patient orientation program designed to assess patient’s overall health prior to surgery - Methods to improve workflow - Quality tools used to establish the critical pathway for developing the APP role
Resources	- Increased staffing allocated; more nurses assigned to the OR to support higher volume of TJR - Good distribution of TJR between all surgeons
Main contextual factors	
- Government funding to increase the volume of TJR - Need for clear objectives at start of fiscal year: “*Cancelling and then rebooking, and ... the hours that must have gone into the scheduling, you know? .... I think it’s important that the thing is clear, or as clear as it can be, at the start of the fiscal year, so that it gets planned out, and then if there need to be adjustments, the sooner we know the better.” (I.4.2)*	
Unintended consequences	
Serendipities	- Greater trust between surgeons and APPs: “*We did some early research looking at level of agreement just to build the confidence. So we really hadn’t had that, it was like a brand new role; but we had all of the tools to show why it would be a good fit, and to gain their confidence. There’s a clear line of communication, everybody knows their roles, we developed algorithms so that the advanced practice physios are integrated into the process.” (I.4.7)* - Improved OR efficiency: *“But you can’t maintain that kind of throughput unless there’s space dedicated, and equipment dedicated, and personnel dedicated to the anesthesia part.” (I.4.2)* - High patient satisfaction (due to better communication from APP) - Better communication with family physicians: *“It’s been great because we’re actually even fostering better inter-professional education with family physician residents.... We just recently had a family practice resident who spent six weeks with us because they deal a lot with arthritis issues, so they were learning from us – it was one of the rheumatology residents [who] spent time with us – because there’s a lot of interaction possible because of the way we’ve aligned our clinics.” (I.4.6)*
Negative consequences	- Work overload for nurses - Increased waiting times and cancellations due to patient preferences - Increased waiting times due to GP resistance or lack of comprehension of the referral system: *I’m not sure that the family doctors understand that what we’re trying to do is get all of the referral information into a central area, there’s a checkbox, a minimal amount of information is required for you to go through that, and if you provide that information, we’ll do our best to get your patients assessed either quicker, or by whom you request in a timely fashion; but it won’t be quickly (I.4.2)*

**Table 7:** Case 5 (Central Canada) - Factors affecting WTMS sustainability and unintended consequences

Organizational factors	
Governance	- Surgeons, staff and middle and top managers aligned toward the same goal: treating people in as excellent a way as possible - Integrated clinical-administrative governance along the continuum of care: “*A balance between staff members that are upstream and downstream must be attempted. The entire staff must feel involved in decision-making because, at each key stage of the continuum, each person has an impact on others. Everyone is interdependent.” (I.5.3)* - Committees to coordinate strategy and to sustain ongoing work
Culture	- Culture of innovation and managers’ sense of responsibility to the population and patients: “*We have a desire within the organization to see how we can improve this continuum of care, to build on each program skill to better serve clients.” (I.5.3)* - Team cohesiveness, staff and managers (middle and top): “*This is a solid team. Our orthopedists are involved, too. Of course, the team is very united, nursing, orderlies, nurses, as well as orthopedists.” (I.5.2)*
Methods and tools	- Letter of non-availability; standardized preadmission form - Shared software application used to plan OR surgical activities - Computerized care plan developed for post-operative care - Dashboards and audits designed to monitor wait times and volume of activity: “*We realized that some of our patients could go home after two days. So then we thought, ‘Well, we’re going to try to reduce the duration of our stays down to 3 days, making it the new target that we want to look at.’ We had meetings every month, with data from our archives. Archives provide us with the percentage of returns to home, which we compare with our target…. Then, we try to see how we can make things better in order to really manage to decrease stay durations, and increase returns to home. If there is a decrease, we analyze why.” (I.5.6)*
Resources	- Increased staffing (nurses and physical rehabilitation technicians) allocated: “*So, having consolidated the examinations in one day, collaboration is required ... for example, we had privileged access for blood tests, for radiology, for the electrocardiogram, ... So we notify the electrocardiogram that, that day, they will have eight orthopedic patients, so they can plan to have enough staff. It means having Unit Leaders who allocate resources.” (I.5.1)*
Main contextual factors	
- Significant government funding to increase the volume of TJR	
Unintended consequences	
Serendipities	- Strengthening of collaboration between clinical and administrative management throughout the establishment: “*The goal, I always say, is to engage them [doctors]. And it’s all about the power of influence. Over time, in surgery, we managed to have a great power of influence because they saw it as a win-win. We were with them, and I had a stake in them operating more; and for me, we were all in this together. You have to understand that there is a connection that happens between the administration and the medical team, through which we have a common goal. And that’s, for me, one of the cornerstones of success.” (I.5.6)* - Better cost control (for prosthesis purchasing) thanks to improved collaboration

### Case 1: Atlantic Canada (non-sustainable)

**WTMS**. In 2008, the Ministry of Health approved a contract between a regional tertiary-care teaching hospital and an ambulatory clinic at another regional hospital to perform additional TJRs over the following year. The same year, a provincial Orthopedic Assessment Clinic (OAC) was opened at the hospital to develop and implement a central intake process for the “next available” surgeon, and to improve access for patients waiting to see an orthopedic surgeon. This pathway began with a newly developed form for community physicians to refer patients to the OAC’s central referral office. At the OAC, patients were met by a nurse case manager for a health assessment, then a surgeon conducted a surgical assessment to determine the likelihood of success for the surgery. Patients were then booked through a Pathway Healthcare Scheduling (PHS) system and awaited a surgical date. A few weeks before the surgery, patients were educated regarding what to expect as part of the surgery, their expected length of stay (LOS), and what they would need at home after surgery.

In this case, WTMS failed to reduce waiting times to less than 26 weeks within an 18-month period.

#### Factors facilitating or impeding WTMS sustainability

**Governance**. The strategy was mainly supported and initiated at the provincial level, by the Ministry of Health. Within the hospital, the surgical services manager tried to increase nurse involvement in decision-making and on committees to support the strategy, but they showed little interest in taking on responsibility outside of their normal work.

**Culture.** Initially, physician buy-in was quite difficult. Most resisted the idea of a central referral system because they wanted to maintain their own patient waiting lists. Some senior surgeons preferred to review referrals coming in each morning, rather than allowing clerical staff to triage patients and determine the urgency of sending them to the assessment clinic. One interviewee indicated that the generational gap between younger and older orthopedic surgeons made it challenging to shift the culture. Others mentioned the lack of involvement of upper management (e.g., the CEO or VP) in promoting innovation in the hospital.

**Methods and tools.** The government created a public website to inform patients, families, and family physicians of waiting times for TJR at the hospitals in the region. While this should have allowed general practitioners (GPs) to direct patients to hospitals with the shortest waiting time, it did not always work out that way. At the organizational level, the team used the computerized PHS system to book and register referrals. They also used a Discharge Abstract Database containing retrospective data about patients’ LOS by type of condition, to track all patients who came through the hospital. The assessment clinic manager submitted reports on key data to the provincial ministry of health on a regular basis to justify their funding. Despite the new referral system, the lack of a standardized procedure for improving the referral process undermined implementation and sustainability of the WTMS.

**Resources.** The increase in resources allocated by the government provided for renovation of the assessment clinic; for clinic staff salaries; for the PHS system; for a website with patient information on surgery and what to expect after surgery; for incremental funding when the surgery target was exceeded; and for more OR time. Nevertheless, interviewees indicated that the resources allocated by the government were insufficient considering population characteristics and needs, notably with regard to the high proportion of the population with arthritis. The organization also faced unavoidable resource issues. Orthopedic surgeons had to compete with other specialty groups for OR time, and arthroplasty cases were often bumped by more urgent cases. Moreover, occasional nursing shortages made it difficult to maintain optimal OR productivity over time.

#### Unintended consequences

Two positive factors (serendipities) stemming from the WTMS implementation were identified. First, the strategy resulted in the development of new patient education programs as a means to reduce WT. Second, the strategy served as a model for program improvements in other areas of specialization.

An unintended negative consequence was that public awareness increased the demand for orthopedic care at the hospital. Given family physicians and patients observed that the hospital had reduced waiting times, physicians starting referring more patients to that hospital, making it difficult to maintain low wait times.

### Case 2: Central Canada (moderately sustainable)

**WTMS.** This community teaching hospital first decided in July 2007 to create a hip and knee care pathway to standardize patient care provided by nurses, physiotherapists, and orthopedic surgeons. In January 2009, it created its own Regional Joint Assessment Centre, relying on an advanced practice physiotherapist (APP) responsible for triaging and screening patients, and arranging a consultation with an orthopedic surgeon if needed, usually within two to four weeks after receiving the referral. For triage purposes, patients are separated into three groups according to the LOS and level of care needed. GPs and patients may opt for the first available surgeon rather than their surgeon of choice. The APP keeps GPs informed throughout the process, so they can track their patients’ status. Further along the pathway, staff from the rehabilitation centre can refer to patient care orders as indicated by the surgeon before a patient’s discharge. Finally, the APP sees all patients for follow-up assessments after their surgery.

In this case, the WTMS reduced waiting times to less than 26 weeks within an 18-month period, but was not able to maintain this target within the period.

#### Factors facilitating sustainability of the WTM strategy or program

**Governance.** The organization signed accountability agreements with the health region, which specified agreed-upon goals. Consequently, the organization’s funding – and hence their surgical volumes – depended on regular performance reports submitted to the health region demonstrating that their efforts were resulting in reduced waiting times.

**Culture.** Within the organization, the director of surgical services and the APP acted as leaders for change. It was interesting to see that the director, who did not have a clinical background, was able to bring his own accounting and management expertise to bear, while obtaining clinical input from his colleagues. Surgeons exercised authority over the entire team of healthcare professionals and would only become involved in an initiative once they saw the benefit for them and their patients in both the short and long terms. This generated some negative subcultures and a lack of trust, as both nurses and anesthesiologists felt they were working for the surgeons rather than with them. What seemed to bring them together was the influence of the director of surgical services, who, due to his non-clinical background, was impartial and had a neutralizing effect on the tensions between professionals.

**Methods and tools.** The decision support manager and the IT department used a province-wide wait time software system. However, they quickly realized the data collected was of poor quality.

Additional strategies were implemented to improve reporting. A provincial tool was adapted to meet the organization’s needs. To reduce the high turnover rate of clerical staff trained in data management, and surgeons’ resistance to entering data in the system, the health region worked with the team of professionals to improve data collection and interpretation. Despite the health region’s willingness to work with the hospital, there were some areas of tension. First, the health region did not initially support the hiring of an APP. Second, certain staff believed that the way of measuring wait times, according to the 90th percentile,^1^ captured only a small portion of patients and was therefore not indicative of actual waiting times for the 10% of patients who needed to wait over 26 weeks for their surgery.

**Resources.** If the performance data on wait times submitted by the HCO to the health region does not show improvement, the health region can choose to withdraw funding. The HCO team had to argue with the health region that funding to increase surgical volumes initially had the opposite effect of increasing waiting times, as they had to clear their backlog. Significant capacity-related issues were reported. Although there was a sufficient number of ORs (12 OR theatres for seven orthopedic surgeons), OR utilization was not as efficient as it could have been. Solutions such as “double joint days” (where a surgeon has two sets of staff, so that one can set up while the surgeon operates in the other room) were not sustainable due to insufficient staffing – of nurses, in particular – and to the upcoming retirement of a few anesthesiologists. There were additional difficulties around efficient bed management and utilization. Few of the 10 beds located on the surgical unit were reserved for orthopedic patients.

#### Unintended consequences

The one main serendipity identified in this case was an improvement in leadership resulting from the WTM program, which in fact served as a model for other programs to improve referral processes and dialogue with GPs.

During the pre-hospital phase, GPs made double referrals to accommodate patients looking for either their preferred surgeon or the first available surgeon. This practice, which ultimately increased WT, was linked to GPs’ lack of knowledge of the program and resistance to change. Patient preferences, e.g. for the surgeon with the best reputation in the region or for a specific period of the year, also increased waiting times.

### Case 3: Western Canada (moderately sustainable)

**WTMS.** The third case is a regional strategy implemented at one hospital site by a regional health authority (RHA). Of two sites offering elective TJR surgery in the RHA, one was in a community secondary-care hospital specialized in orthopedics involving joint replacements. At this site, a Hip and Knee Institute (HKI) was created in 2009, dedicated to HK research surgery and to pre-habilitation and education programs for patients. The strategy was first initiated in 2005 at the RHA level with a cleaning of the waiting list to ensure information was accurate and up to date. At the RHA, pre-surgical patient management strategies included setting up a regional pre-surgical health optimization clinic staffed by an interdisciplinary team of medical and allied health care providers. The queuing methods implemented to improve wait list management efficiency included assigning patients on the arthroplasty wait list to one of three categories: 1) Ready for surgery, 2) Delayed for medical reasons, or 3) Delayed by personal choice. With such a system, surgeons’ offices can manage surgical scheduling based on patient readiness, while patients can be placed in the queue even if they require medical clearance. All of these strategies were implemented to ensure patient readiness for surgery and booking efficiency, while maximizing surgical outcomes, minimizing complications, and decreasing lengths of stay associated with surgery.

In this case, the WTMS temporarily reduced waiting times to less than 26 weeks, within an 18-month period, but was not able to maintain this target within the period.

#### Factors facilitating the sustainability of WTMS

**Governance.** The strategy benefited from a high level of support from the RHA. Another important factor was that, as the hospital was mainly dedicated to elective orthopedic surgery (75% volume of activity), it was also able to implement a two-room model. The governance strategy focused on ensuring a centralized intake and better distribution of cases/referrals in order to advance the agenda of improved accessibility for patients, better resource utilization, and accountability through an equitable distribution of the surgical workload. As the HKI received a large number of patient referrals for surgery due to its academic reputation, the RHA had to adjust referrals distribution among all orthopedic surgeons and the two hospital sites, for example by calling patients and offering them a new surgeon and hospital/clinic.

**Culture.** Strong leadership was provided by an orthopedic surgeon within the HKI, where all surgeons were doing just TJR surgery. All the surgeons were of the same generation and shared a similar performance culture. WTM sustainability was a challenge for the RHA, because surgeons did not all share common goals concerning the WTMS. At the other hospital site, where the strategy had not been implemented, orthopedic surgeons were doing different kinds of surgeries, were less interested in participating in the program, and did not try to implement specific strategies to increase volumes. An interviewee observed that financial incentives, offered when more surgeries are done, are not enough to implement a strategy; common goals and values are also necessary.

**Tools and methods.** At the RHA level, tools were developed through the centralized intake process. A Patient Access Registry Tool (PART) was introduced to capture data on how many patients were waiting for procedures/consults and how long they were waiting. PART, which was deployed to all surgeons’ offices in November 2011, was intended to reconcile wait lists and help surgeons’ offices better monitor their longest-waiting patients. Another tool developed for the centralized intake process was a standardized referral form to ensure better coordination between GPs and specialists.

**Resources.** RHA special funds allocated to the HCO for the purpose of increasing joint surgery volumes. Although no part of the funding was specifically allocated to staffing, these resources were used to hire pre-rehabilitation clinic staff and two clinical assistants for the operating rooms. With one surgeon operating in two rooms (the two-room model/strategy), the HKI was able to double its case output from four to eight cases a day.

#### Unintended consequences

The program served as a model for other programs to improve their referral process and dialogue with GPs.

A couple of negative unintended consequences were mentioned. First, patients did not understand the referral process very well due to the lack of communication. In addition, GPs did not always follow the appropriate referral process. Another major undesirable and unanticipated negative consequence was an increase in waiting times and a bottleneck within the HKI, due to patients’ preferences regarding timing and surgeons, and due to the Institute’s strong reputation. Indeed, despite the fact that there were three sites, the vast majority of patients asked to be treated at the HKI and were willing to wait. The fact that not all three sites implemented the same strategy resulted in increased waiting times for the most effective site.

Permanent changes to strategies causing variations in the program’s outcome had both positive and negative impacts on patient satisfaction, depending on the timing of the implementation. It also led to staff exhaustion.

### Case 4: Central Canada (sustainable)

**WTMS.** In May 2007, this health region created a Steering Committee to implement a comprehensive hip and knee replacement program for effective management across the complete continuum of care for patients needing TJR. It funded a new Intake and Assessment Center model that incorporated an electronic referral tracking system, timely assessment by a APP emphasis on patient choice and empowerment, selective referral for specialist care based on evidence-based data, follow-up care post discharge, and community partnerships to encourage healthy living for patients. The HCO in this case is a satellite tertiary-care teaching hospital center operating at a different location than the main regional hospital with which it was merged ten years previously. The main hospital offers acute medical and surgical care to patients in a metropolitan city in Ontario; the satellite hospital focuses primarily on hip and knee surgeries and accepts patients from outside the city.

In this case, the WTMS resulted in all patients waiting less than the 26-week benchmark for TJR, for at least 6–12 months within an 18-month period.

#### Factors affecting WTMS sustainability

**Governance.** The program had strong leadership support from the hospital’s CEO and all members of the senior management team. Many members of the HCO involved in piloting the model showed strong organizational leadership and dedication to their work from the outset of the program. A sense of involvement, collaboration, and mutual trust was evident among staff.

**Culture**. The hospital, which has its own rehabilitation unit, is entirely dedicated to hip and knee care, so there were no capacity constraints or ‘bed blockers.’ It only takes patients in need of simple hip or knee surgery, with few co-morbidities, while patients with complex needs are diverted to other hospitals in the area. As all healthcare professionals working in this satellite hospital had been there a long time, there was a stability and cohesiveness among them. Many stated their intention to remain the best and to be creative and innovative. While implementing the initiative, they exercised leadership in the community by sharing their innovation and commitment. The Ministry named the hospital a Centre of Excellence for Hip and Knee care. The team’s stable, unified culture, and sense of commitment to the work being done, all contributed to the team achieving consistently low waiting times and help explain why this case was categorized as sustainable.

In addition, the chief of surgery, an orthopedic surgeon, essentially convinced his physiotherapist and program director colleagues to pilot the APP model before funding from the Ministry had even begun. This surgeon was used to dealing with his surgeon colleagues and had led a lot of change through other programs he had implemented internally. He started using the model in his practice, and this influenced other surgeons to start doing it as well. The team also ensured they involved all levels of care in the decision-making to avoid obstacles during the implementation phase. Many interviewees commented on the abundance of committees created to support the strategy and to sustain the ongoing work.

The APP model was implemented very gradually, so the APPs could get used to their new roles and the surgeons and other team members could get used to the new way of functioning. In addition, the program director explained that once the team was able to demonstrate that the model of care was useful and making progress, they gained support from people they reported to.

**Methods and tools.** The central intake clinic serves as a single point of contact for patients and referring physicians who need to access care for hip or knee arthritis at any of the six regional hospitals. A standardized request form for consultation was developed to support the process. Practice-based development programs were designed, and formal training was provided for the APPs. This model of care has encouraged many local partnerships, notably with the province’s College of Physiotherapists, which helped develop the APP role. It has also encouraged better communication with family physicians referring patients to the clinic. The team used different quality tools, such as the Plan-Do-Study-Act cycles and the Participatory Evidence-based Patient-focused Process framework, to establish the critical pathway for development of the APP role. The assessment center provides patients a timely assessment of their hip or knee problem, along with education and advice. If patients are surgical candidates, the nurses at the pre-operative clinic assess them. Patients are usually seen at the assessment center two weeks before their surgical consultation. The patient pre-operative orientation program is designed to assess patients’ overall health prior to surgery. The interdisciplinary assessment form is a tool to collect bio-psycho-social information on patients. Classes were developed to provide patients with the information they need before and after their surgery. With regard to the surgical and post-operative processes, two programs were created – the regional anesthesia program and the acute pain program. Methods to improve TJR workflow were also developed: anesthesia block-room opportunities, pre-admission improvements, a two-OR model, and an OR scheduling algorithm to improve the bed count. Care pathways were also developed to guide and standardize patient care after surgery. Patients could either do inpatient rehabilitation, outpatient rehabilitation, or go home with home care services provided to them according to their progress. Patients are seen in the post-operative review clinic by an APP, six weeks after their surgery.

The satellite hospital received funding from the health region to develop the referral tracking system (RTS), which reports patient wait times. This system, developed by the satellite hospital’s IT department in partnership with its care team and a division of the Health Ministry, is now the provincial system, used by the six other regional hospitals. This is another example of the team’s innovative culture. A leadership team meets monthly to review the data and track the team’s progress. The region has been reviewing the data on a monthly basis to assess the hospital’s performance, as stipulated by the accountability agreement with the hospital. Ultimately, a “toolkit” was produced and offered as a knowledge transfer tool to hospitals wishing to improve their surgical performance.

**Resources.** The main helpful contextual resource factor during both the implementation and sustainability phases of this strategy was government funding to increase surgery volume. This funding helped cover the costs of OR and inpatient care. While the methodology and the transparency of volume allocation have improved, it has remained difficult for hospitals to adjust to new volumes on a year-to-year basis, especially if there are mid-year changes. In that regard, there was tension between the hospital and the health region. Hospital managers stressed the importance of having clear objectives before the start of the fiscal year to plan their work more effectively. In addition to the APP, the team has implemented other new roles, such as nurses in the OR, registered nurse first assistants who effectively function as assistants to the surgeon throughout a patient’s surgical experience, and nurse practitioners who help on the pain team. The team was concerned about whether the health region would withdraw funding. At the time of the site visit, their surgical capacity had reached about 2100 per year. Some surgeons mentioned how big a change it was to have their OR time nearly doubled. They persevered because they associated it with positive outcomes. Since they were putting more surgery lists back into the system, these were shared among all arthroplasty surgeons, allowing them all to do more surgery. They were never bumped for other types of surgeries, as there were four OR theatres for ten orthopedic surgeons.

#### Unintended consequences

Several serendipities were identified in this case. First, the WTM program significantly improved relationships and established trust between surgeons and APPs. Second, it resulted in improved efficiency due to the OR being used exclusively by the TJR team. Finally, APPs became an essential part of the WTM program, improving communication with patients, which resulted in a high level of patient satisfaction.

One negative consequence was that nurses were overworked. Another was increased waiting times for the referral process due to GPs’ resistance or lack of comprehension of the mechanism, which meant they did not always provide sufficient information to the intake center. Patients’ choices also affected both wait times and efficiency, for example when patients preferred to have surgery done within a specific period or by a specific surgeon.

### Case 5: Central Canada (sustainable)

**WTMS.** The strategy implemented in 2007 was based on a central management mechanism, which involved creating an Access Office in charge of coordinating surgical operations, waiting lists for preadmission to postoperative rehabilitation, and hospital stays. The strategy, which provided for budget allocations to institutions from the Ministry of Health to increase the volume of TJR, was based on consultation and coordination of all health professionals and administrators involved in the patient’s clinical pathway. For preadmission, preoperative examinations were scheduled together on the same day and organized by grouping eight total hip and total knee replacements simultaneously to optimize management of preoperative progress and facilitate coordination with the various examination services. In addition, a group teaching session and visits to the patient’s home helped plan for their return home. OR surgical activity planning was significantly reorganized. The number of operating time slots was increased, and clinical planning software improved efficiency. Standardized computerized daily care plans were put in place for the postoperative phase, to fostering better functional recovery for patients and shorten their hospital stays.

In case, the WTMS resulted in all patients waiting less than the 26-week benchmark for TJR, for at least 6–12 months within an 18-month period.

#### Factors affecting WTMS sustainability

**Governance.** As a condition for the significant financial incentives offered by the MSSS, the institution was asked to review its organization to increase productivity. The institution uses an OR Coordinating Committee that represents clinical and administrative co-management. The committee, consisting of all health professionals and administrators/managers directly involved in the process, meets every month to discuss the care continuum, including operating room activities. The OR Coordination Committee set up agreements between the preadmission clinic and internal medicine, radiology, laboratory, and physiotherapy/rehabilitation services to plan preadmission days in orthopedics (including TJR). With this consultation structure, information regarding patients’ progress (change in the average duration of stay, review of postoperative protocols, etc.) is communicated regularly. Information provided to patients during education sessions can be updated to promote effective preparation for the patient’s return home.

**Culture.** From a management perspective, there is a desire to best serve the population according to its needs and to promote coordination between different stakeholders to ensure a continuum of care. Another cultural factor was the strong cohesion of not only the medical team, but the whole team, including management and managers. All were working toward the same objectives.

**Methods and tools.** Several methods and tools were developed, such as a letter of non-availability, which is systematically sent to unavailable or unreached patients who have already been sent offers twice, and a standardized preadmission form, which facilitates the management of medical priorities (P1, P2, P3, P4) and ensures that the preadmission clinic is well aware of priority levels.

OR surgical activity planning was also reorganized. By increasing the number of operating slots and tracking surgeon availability systematically using a shared software application, the operating schedule can be set up at least one month in advance. Dashboards were set up to monitor waiting times and volumes of TJR activities, which are compared every two months against anticipated targets. Using this systematic monitoring mechanism, operating schedules can be adjusted based on anticipated business volumes, thereby improving waiting time management. On the post-operative side, computerized care plans for each postoperative day (D1, D2, D3, D4) were developed to support functional recovery of patients by systematically monitoring their progress. In addition, regular audits are carried out to ensure that data is collected systematically for all patients. An expanded work panel, bringing together all professionals involved in postoperative care, periodically checks the average duration of stays and the percentage of returns to home.

**Resources.** Two staff (one nurse and one secretary) were allocated to the preadmission clinic to coordinate requests, monitor list management, and follow up in coordination with the team. Additional staffing (internal medicine specialist, radiologists, etc.) were planned for days of preoperative examinations to meet the increased volume. Physical rehabilitation technicians were also hired to help physiotherapists systematically monitor postoperative patients.

#### Unintended consequences

Two positive effects were mentioned. First, the successful collaboration between doctors and managers strengthened clinical and administrative management within the institution. Second, this collaboration also led to a discussion of the cost of hip prostheses, so that doctors agreed on hip implants that are medically relevant and affordable. As a result, fewer prosthesis types and fewer different models are purchased.

One negative consequence was that reorganization increased waiting times for other non-priority surgeries such as arthroscopy. This sometimes creates conflicts between orthopedic surgeons and other surgeons.

## Study limitations

One of the foremost limitations of this study was the difficulty in finding non-sustainable cases. Three of the organizations that we approached refused to participate, as they were reluctant to share negative experiences. One establishment that represented a successful case also refused our offer, which was less understandable. While we had hoped to have two cases per category, we were unable to find two non-sustainable cases willing to report. Another limitation was the retrospective qualitative design, which could be affected by individuals’ memory biases.

## Discussion

This research analyzes factors that can help explain differences in the ability to maintain wait times below the national benchmarks and examines whether implemented strategies have had unintended consequences. Concurrent analysis of both of these dimensions has not been covered in the existing literature.

Our study sheds light on factors that might explain the success of certain strategies. We saw that there were significant differences between cases in the three categories (sustainable, moderately sustainable, and non-sustainable). Apart from financial incentives implemented by provincial government, the sustainable cases had the following common characteristics at the organizational level:

1. A WTMS that took into account the whole care continuum, from the pre-clinical stage until the return home;
2. Strong clinical leadership uniting upper/middle management and physicians in a common purpose;
3. Committees to coordinate strategy and to sustain ongoing practices, processes, and outcomes (i.e. improved wait times for TJR); and
4. An organizational culture based on trust and innovation.

The orthopedic teams that we studied redesigned care processes to improve efficiency and shorten patients’ length of stay. To this end, all five sites developed and implemented a clinical pathway. These clinical pathways are commonly used as one of the methods for structuring care processes in HCOs [26]. We observed that cases 4 and 5 had dedicated the most time and effort to developing and implementing their care pathway. To achieve this, they set up governance committees to coordinate the WTMS and to monitor the processes and practices implemented as well as the strategy’s outcomes (i.e. improved waiting times for TJR). Such governance structures are essential in any change initiative [27]. Moreover, work methods and tools were developed to evaluate and to systematically follow patients at the different stages of care (from pre-op to post-op, including rehabilitation). Among the tools and methods common to the sustainable cases, we note tools/systems for wait-time tracking, standardized forms for medical consultation, and tools/methods to improve planning for patient pre-admission, surgical activities, and bed management. Strong leadership, cohesiveness/collaboration between managers and clinicians, and an organizational culture geared toward innovation, all made it possible to develop coherent strategies along the care continuum. These cultural factors are also essential components for access sustainability. Indeed, sustainability visions and strategies become internalized as individuals consider what these changes will mean to them personally [28, 29].

The less sustainable cases (unsustainable and moderately sustainable) had implemented strategies that took into account the care continuum, but took place in contexts where not all the above factors were present. One of the factors most often missing was shared medical leadership. In both cases, a physician led the drive for change, but was confronted by resistance from colleagues or other professionals. Another important limitation concerned resource utilization and management. In cases 1 and 2, nursing shortages and the existence of competition between surgeons for OR time prevented the HCOs from doing more TJR. One of the moderately sustainable cases (case 3) also showed the limitations of implementing a new model on one site while pursuing orthopedic activities at other sites, without redistributing the caseload. Such an approach prevented the strategy from achieving its optimal effect. In fact, if the model had been fully adopted, the Institute would have been the only site where elective knee surgery was performed, and the other sites would have performed the more difficult cases. Such a model might have resembled that of the sustainable Ontario case study. In the one non-sustainable case, it was clear that many of the factors listed above were absent, including the absence of upper management involvement and support for WTMS.

These results are consistent with other studies in the literature regarding factors that facilitate or impede the success of WTMS [22, 30], but our study provides further detail and sheds light on factors that are more significant than others.

### A need to sustain WTMS in a systemic and scientific way

Although the organizational factors mentioned above are essential to achieving sustainability in WTMS performance, a more systemic view has to be adopted to better understand how waiting times are maintained over time. Our results are consistent with the conclusions of other studies on sustainability of waiting time management, which highlight the importance of considering the broader context of any new program or change strategy [31, 32], including WTMS [22]. The most important contextual factor identified concerned recurring financial resources allocated from provincial or regional levels to WTMS at the organizational level. Having a clear idea of their annual funding allows teams to plan their upcoming workload and the required resources more effectively. Greenhalgh et al. [31] explain that dedicated and consistent funding for a strategy increases the chances of that strategy being not only adopted but also sustained over time. In fact, this was a problem encountered by each of the three less sustainable cases from the outset of their strategy implementation.

A culture of measurement is another key element, ensuring that data is used with the aim of improving the efficiency and effectiveness of strategies and processes [29]. Although the importance of measures at the organizational level was mentioned in all cases, the importance of developing methods and tools at the provincial level, a key element identified in the literature [7, 22], was only illustrated in two of our five cases (cases 3 and 4). Indeed, Canadian provinces have quality councils that could serve as a resource, helping initiatives to select relevant tools for implementing their strategies.

As far as change implementation strategies are concerned, our interviews did not reveal any that were based on the literature, except in case 4, where the WTMS was based on Plan/Do/Check/Act methodology that provided a structure for implementing changes to improve the quality of the process/care pathway [33]. For the other cases, it appeared that organizations reacted to the financial incentives that were put in place without giving sufficient consideration to wider issues, such as the phases of change management or unexpected consequences that might arise. Although it was not stated explicitly, the two sustainable cases were the ones that actually used project management strategies. In both cases, the evidence pointed mostly to strategies based on trial and error or on quite intuitive but structured models of management. In the unsustainable and moderately sustainable cases, there was an apparent lack of project or change management at both the strategic and organizational levels – better use could have been made of scientific tools and knowledge in this area. In fact, mobilizing change skills can help ensure the sustainability of a new strategy or program over time [32]. The concept of sustainability implies that a change initiative contributes to reducing a problematic situation over the long term (e.g., waiting times for TJR), without causing unacceptable and unintended consequences at the organizational and contextual levels [34].

### Managing WTMS to avoid or mitigate undesirable consequences

Results showed that WTMS resulted in both desirable and undesirable unintended consequences. Without a strategic and anticipative global vision of access management at organizational and higher levels, the strategies implemented tended to produce negative unintended consequences that compromised their long-term sustainability [22]. Regardless of the type of strategy and the success of the case, all of the strategies led to relatively similar serendipities and negative consequences.

The recurrent negative consequence that emerged for the majority of cases was an initial increase in TJR waiting times, due to public awareness of the WTMS at the specific facility and/or patients’ preferences for certain surgeons or periods of time. For case 5, WTMS resulted in increased waiting times for other non-priority orthopedic surgeries. For the less sustainable cases (cases 1, 2, and 3), this initial increase in waiting times persisted over time.

Among the positive consequences (serendipities) for the majority of cases, the implementation of WTMS in TJR programs provided a model that led to review of operational procedures for other areas of specialization.

### Implications for decision-makers and managers

These case studies provide guidance for provincial and regional decision-makers, and HCO managers, on factors that affect the sustainability of strategies targeting the reduction of wait times for TJR. To date, there is no evidence on how managers can help to sustain WTMS over the long term while eliminating or mitigating negative consequences [35].

To be sustainable, WTMS need to result in greater synergy between contextual levels (provincial or regional) and organizations. For managers at the contextual level, it is important to be sensitive to HCO realities when establishing objectives. For example, funding an increase in surgical volume does not necessarily lead to a reduction in surgery wait time. Funding should be predictable and recurrent from one year to the next. It is also important for decision-makers at provincial or regional levels to offer some strategic guidance to organizations, by developing more specific WTM methods and tools. At the organizational level, the results suggest that close cooperation between managers and physicians is needed to sustain WTM improvements. Managers also have to ensure WTMS are integrated into organizational structures. Lastly, managers at the organizational level should be vigilant with regard to the unintended consequences that a WTMS in one area can have in other areas of care. A more systemic approach to sustainability can help mitigate undesirable unintended consequences.

## Conclusion

This study highlights important differences in factors which help to achieve and sustain waiting times. To be sustainable, a WTMS needs to generate greater synergies between contextual-level strategy (provincial or regional) and organizational objectives and constraints. Managers at the organizational level should be vigilant with regard to unintended consequences that a WTMS in one area can have for other areas of care. A more systemic approach to sustainability can help avoid or mitigate undesirable unintended consequences.

## Additional file

Additional file 1: Semi-directed interview guide Interviews with people involved in WT management for HKR in Regional Health Authorities, Hospitals or Clinics. qualitative data. (DOCX 23 kb)

## Abbreviations

APP: Advance practice physiotherapist
GP: General practitioner
HCO: Healthcare organization
LOS: Length of stay
OAC: Orthopedic Assessment Clinic
OR: Operating room
PHS: Pathways Healthcare Scheduling
RHA: Regional health authority
TJR: Total joint replacement
WTMS: Wait Time Management Strategy

## Footnotes

1 The 90th percentile indicates the point at which 90%, or 9 out of 10 patients, received their surgery, and the other 10% waited longer. For example, 90% of patients in need of a TJR underwent surgery within 26 weeks, and the remaining 10% waited longer than 26 weeks.
