# Supplementary material for: Wait time management strategies for total joint replacement surgery: sustainability and unintended consequences
Source: BMC Health Serv Res. 2017 Sep 7;17:629. doi: 10.1186/s12913-017-2568-6 (PMC5590149; doi:10.1186/s12913-017-2568-6)
Supplement: Additional file 1: — Semi-directed interview guide Interviews with people involved in WT management for HKR in Regional Health Authorities, Hospitals or Clinics. qualitative data. (DOCX 23 kb) [file 12913_2017_2568_MOESM1_ESM.docx]

**Semi-directed interview guide**

**Interviews with people involved in WT management for HKR** **in Regional Health Authorities, Hospitals or Clinics**

**Background**

1. When did you start working in health care?
2. What is your educational/professional background?
3. What organization and sector do you work in?
4. What are your title and your functions?
5. What is your role in WTM strategies for total joint replacement surgeries?

**Organization situation**

- What is the organization’s financial situation?
- recruitment

**WTM Strategy**

1. Can you describe the strategy that was implemented in your organization to reduce wait times for HKR?
   1. How did the strategy originate? Did it start out as a provincial, a regional or a local initiative?
   2. In what context was the strategy implemented?
   3. At the time that the strategy was implemented, what were the major causes of the problem? Examples: inadequate resources, a lack of coordination, a lack of governance at the provincial/regional/local level, a lack of leadership, the wrong management tools
   4. What were the motivations for fixing the problem?
   5. Has the WTM strategy changed from its beginnings to now?

(for the interviewer: description of the WTM strategy and classification of the means used to reduce wait times)

1. Can you validate the impact of the strategy on the waiting time between the decision to treat and surgery?

(For the interviewer: statistics that we have collected and statistics that we have asked of the WTM coordinator)

1. What would you consider a successful WTM strategy? Would you characterize this initiative as a successful strategy, a moderately successful strategy, or a strategy that should be improved? Why?

(For the interviewer: our own definition to discuss with the interviewee)

1. How do you personally define a sustainable WTM strategy?

(For the interviewer: our own definition to discuss with the interviewee)

**Implementation phase**

1. When did the strategy’s implementation process begin?
2. What time frame do you feel corresponds to the implementation phase? Are we still in that phase?
3. What factors enhanced or inhibited the implementation phase?
   1. What governance factors?
      1. Were the Board and the CEO involved in designing and implementing the WTM strategy?
      2. Who was responsible for the implementation of the strategy?
      3. Were/are you involved at the project’s implementation phase? If so, how?
      4. What type of governance measures were put in place for implementing the strategy? (special committees, etc.)
   2. What cultural factors?
      1. How would you categorize the culture of this organization? (hierarchical / rational / group / developmental)
      2. Were physicians involved from the beginning?
      3. What were physicians’ roles and responsibilities regarding the initiative?
   3. What resource factors?
      1. Were specific resources dedicated to the project: human, financial, infrastructure, information resources?
      2. In the implementation phase, did you have access to reliable information to help manage wait times?
      3. Has the hospital conducted its own studies to measure the impact of WTM strategies on waiting times? Are reports available?
   4. What tool factors?
      1. Were specific tools or procedures used?
      2. Did you use operational research, prioritization tools, an organizational care process, the patient pathway?

**Sustainability phase**

1. Were new conditions necessary to sustain this strategy?
2. Did you receive resources earmarked for sustaining the initiative, either from outside or inside the organization?
3. Did you have to change the governance of the WTM strategy in order to sustain the strategy?
4. Did factors that affected the implementation process, change in the sustainability phase?
5. Did you face challenges in maintaining the WTM strategy’s impact on waiting times?
   1. If so, what did those challenges lead to?
      1. E.g.: a difficulty to maintain motivation around the strategy
   2. What challenges were identified?
      1. Inadequate resources, a lack of coordination, a lack of governance at the local/regional/provincial level…

**Unintended consequences related to the implementation of a WTMS in a HCO (positive and negative)
 
Different dimensions that can be touched by the introduction of a WTMS.
 
*The relationship with primary care***-        Did the WTM strategy affect the relationship between primary and secondary care specialists? If so, was the impact positive or negative?
-         Did this cause tension between the primary care providers due to the fact they had to re-evaluate how they referred patients?

-        Did that force them to focus on the wait time issue when they had many others to worry about?

- Was there a lot of resistance to change?
 
***At the clinic level***-        Has the way that surgeons, nurses and physiotherapists work together changed?

***At the OR level***-        Did the WTM strategy distort priorities in the OR? In the emergency room?
-        Did problems of security emerge during the implementation of the WTM strategy?
-        Did the relationship between orthopaedic surgeons and other surgeons change?
-        Did the strategy improve the relationship between surgeons and anesthesiologists?
-        Did the strategy improve the relationship between surgeons and anesthesiologists?
-        Did the strategy improve the relationship between the different professionals involved?
-        Did the strategy create a need to review the organization of the OR in order to maximize surgeons’ OR time?
-       Has this stressed the health care providers and other members of personnel in the way they work?  If so, how did they manifest their resistance to change?

***In the emergency room***-        Have scheduled OR times affected emergency surgeries?
 
***On preparation for surgery***-        Did the strategy impact the way staff prepares patients for surgery? If so, how?
-        Has this increased the level of anxiety for patients who have less time to prepare for surgery?

***On patients’ security***-        Since the WTM strategy was implemented, have more or less medical errors occurred?

- Since the WTM strategy was implemented, have more or less nosocomial infections?
   
  ***Appointment-making within the Organization***-        Has the strategy increased the work of personnel in charge of scheduling appointments?
   
  ***On hospitalization services***-        Are beds on surgical wards reserved for orthopedic surgeries? If so, has reserving these beds sometimes caused excess capacity?
  -        Has the strategy obliged hospitalization services to be restructured in order that the hospital can keep receiving patients? Has this had a positive or a negative effect on bed management?
   
  ***On the post op rehabilitation***-        Has this had an impact on work relations between surgeons, nurses and other colleagues (e.g. physiotherapists) in regard to work organization?
- ***On the entire organization***-        Were budgets modified, taking funds from certain programs to finance the WTM strategy? If so, which programs were affected? What were the consequences?
  -        Have the functions of all professionals who care for patients along the continuum of care been clearly defined?
  -        Has the WTM strategy for HKR encouraged other programs to look at what was done for THK surgeries, in order to reorganize their own program? If so, could you give an example?
   
  ***On stakeholders***-        Has the strategy changed the relationship between the ministry and other partners? (e.g., rehabilitation centers, orthopedic assessment clinics)
   
  ***On patients***-        Has this changed the patient’s experience overall? If so, positively or negatively?

 
Can you think of other dimensions or effects, either positive or negative, that have not yet been discussed here?

Prior to implementing the strategy, did actors consider that implementing this WTM strategy could cause unintended consequences?

- If so, how?
- If not, why not?

**Conclusion**

1. Reflecting on the helpful and unhelpful factors discussed here, which factors do you think were the most crucial to implementing and sustaining the WTM strategy in your organization?
2. What were the WTM strategy’s principal unintended consequences, both positive and negative?
3. What do you think others could learn from your experience?
